# On the Feeding of Dogs in Health

**Published:** 1901-03

**Authors:** Cecil French

**Affiliations:** Washington, D. C.


// THE JOURNAL

OF

COMPARATIVE MEDICINE AND VETERINARY ARCHIVES.

Vol. XXII. *MARCH, 1901.* No. 3.

### ON THE FEEDING OF DOGS IN HEALTH.

By Cecil French, D.V.S.,
WASHINGTON, D. C.

A few months ago a well-known kennel journal requested me to address the principal American breeders of dogs, with the object of obtaining their opinions on the subject of a dietary for dogs. Breeders were selected because they are usually men of large experience, whose business it is to have their animals in good condition. The responses were very numerous, having been sent in from all parts of the United States, Canada, and Mexico. They were all carefully tabulated, so that the reader might be presented with a synopsis of the opinions which showed the greatest conformity to one another. It must be remarked, however, that in a few instances the answers have exhibited a diversity of opinion almost equal in degree to the extent of area from which they were received; but the apparently conflicting ideas came from owners of animals kept under widely different conditions, which goes to show that no hard-and-fast lines can be laid down in the matter of canine gastronomy.

These opinions of the professional dog-feeder are offered to the practitioner in the belief that they will be appreciated and be of some assistance whenever his advice is sought on the matter. The list of questions and the general opinion on each one is noted below, and at the same time a further contribution is offered in the shape of a few remarks from the stand-point of various experiments and observations on the subject of feeding and assimilation in the dog which from time to time have been carried out under the supervision of certain scientific authorities. I am indebted to *Bulletin No. 45 of the Department of Agriculture*, prepared by Drs. Atwater and Langworthy, for citations of the experiments and conclusions referred to.

*List of Questions.*

1. How many times daily do you feed puppies?
2. How many times daily do you feed adult dogs?
3. Do you feed milk? Raw or boiled?
4. Name in order the farinaceous foods upon which your dogs thrive best (oatmeal, cornmeal, etc.)?
5. Do you feed meat, and what quantity? Raw or cooked?
6. Do you use commercial hard-baked dog-foods? If not, state objections.
7. State if you use vegetables of any kind and in what quantity.
8. Mention anything further on this subject that may occur to you as interesting.

In answer to question No. 1, the majority of the breeders of small and medium-sized dogs favors three, or sometimes four, times daily, while those who handle the larger breeds, such as English mastiffs, St. Bernards, and Great Danes, almost unanimously regard four, and even five, times as most conducive to thrift and rapid growth.

Question No. 2 has drawn forth the almost uniform opinion that twice is sufficient for adult animals, consisting of a light meal in the morning and a heavy one at night. A few do not consider three times superfluous, and others owning packs of hunting breeds believe in feeding but once, excepting in the case of stud-dogs and in-whelp and nursing bitches, to which two full meals are given.

Some interesting light is thrown on the question of the frequency of feeding by the conclusions at which Kolpakcha arrived after he had made a series of experiments. Protein is the most important element of nutrition, and is a source of energy in addition to serving for the building and repair of tissue. Any assimilable proteid when taken into the organism serves three purposes: A portion is broken down in furnishing energy for carrying on the work of the organism and the remainder becomes "stored" in the body for future emergency. This "stored" protein later becomes organized tissue protein or an actual part of the body cells, provided the conditions are favorable. When the food contains a sufficient amount of protein or albuminous material very little "stored-up" protein in the tissues is drawn on, since it is protected by the protein of the food. This tendency of the organism to make use only of the consumed protein and not to draw on the stored-up is most noticeable under the following conditions: (1) When the income of protein in the food is excessive and (2) when the daily quantity of food is consumed at several different times. Thus the problem of nutrition comes to be the protection of the tissues from destruction and not the destroying and rebuilding of the same, and the nutrients consumed must serve for the production of the energy of the organism.

It is doubtful whether the dogs of many of the kennels in this country receive more than two meals daily, but the results of the experiments above referred to are of sufficient interest to suggest the practical trial of the "little and often" method of nutrition.

The next question, in relation to milk, brought out the fact that nearly all the dogs reported on receive a daily supply of milk, varying in nutritive value from skimmed to Jersey. The opinion as to the raw or boiled state of the same was about equally divided, but perhaps slightly in favor of the raw for adults and the boiled for puppies. All preferred to boil the milk when the bowels were at all in a loose condition. One answer stated that sour milk or buttermilk were excellent vermifugal agents. Experiments have shown that it is possible to maintain dogs in nutritive equilibrium on an absolute milk diet. For puppies I have found sterilized milk exhibits less tendency to production of intestinal complaints than raw.

Question No. 4 seems to have aroused the greatest variety of opinion. Indeed, some of the writers object to any kind of farinaceous food. Many use whole-wheat bread. Most use cornmeal, but some of these only in winter. Next in favor is oatmeal, with middlings close behind. Rice is strongly advocated by others.

It is difficult to comprehend why there should be any objection to farinaceous food forming part of the dog's ration. While the dog is classified anatomically as a carnivorous animal, and in his wild state undoubtedly is, yet mankind has so domesticated him that he thrives well under an omnivorous diet. A largely representative or purely bread diet is very poorly assimilated in the dog, and no dog can be maintained in good condition on the same. The animal is unable to assimilate sufficient proteid material to serve in the building and repair of tissue and the supply of energy. Beside being composed largely of starch, which therefore cannot form a complete diet for flesh-eating animals, bread feces ferment very readily, and it is believed that the fermentation causes intestinal movements which hasten the excretion of the feces and thus hinder the intestinal absorption of nitrogenous material.

With regard to Question No. 5, it appears to be the general opinion that a certain amount of meat should be given, the majority advising that it be cooked together with the other food. A goodly number believe in feeding it altogether in the raw state, and nearly all feed raw meat once weekly for a change. Many include bones.

Bischoff and Voit found that in order to nourish a dog with meat only so that flesh or fat is not lost from the organism, it requires a considerable quantity of meat, varying from one-twentieth to one-twenty-fifth of the weight of the dog. If less than that is supplied, tissue and fat from the organism will be used up. If more meat is supplied for one day than is necessary for nourishment the excess is stored up. On the following day the same quantity of meat does not suffice to produce the same gain, but all will be utilized. A further gain of muscular tissue can be brought about only by a combined increase in the quantity of food consumed. When a maximum consumption is reached the dog will not eat more and loses weight rapidly. The quantity of meat which the dog needs to cover losses sustained and to gain flesh is always decided by the quantity of body tissue. If the dog has a large quantity of muscular tissue he needs more food than when he has little flesh, and if he gains largely in muscular tissue he must consume an abundant diet of meat.

The consumption of fat from the body can be prevented by consuming fat in some form in the food. With an abundance of fat in the diet it is also possible to gain fatty tissue. The consumption of fat reduces the amount of proteid material used up in the body, so that only one-third to one-fourth as much meat need be consumed with fat as where meat only is fed. For instance, a dog weighing eighty-five pounds while at rest can be maintained in a state of nutritive equilibrium on three and one-half pounds of lean meat daily. The same dog can be kept in the same condition on less than three pounds of lean meat when fat to the amount of five or six ounces is supplied. In place of fat, sugar and starch may be used, since they act in the same manner as fat. When meat and sugar are eaten the subject will gain in weight more rapidly than when meat alone is fed. Starch can be supplied in the form of bread or cereals.

In view of these facts we must arrive at the conclusion that while dogs can be kept in condition on a purely meat diet, provided they have the requisite amount of exercise to keep their digestive and excretory organs in good working order, it is better to combine this article with cereals, particularly as these animals are more or less restrained from taking the exercise which their lives, under natural conditions, would secure. In my own kennel and infirmary I have found the best ration to consist of stale bread and meat, about three parts of the former to one of the latter.

As to the feeding of bones, I am opposed to it. Most of the dog-books and journals advise that bones should be given "in order to clean the teeth." How a dog can clean his teeth by gnawing on a bone is beyond my comprehension. That which constitutes uncleanliness of the teeth is the accumulation of tartar. Tartar is invariably found deposited in between, at the posterior surfaces, and around the necks of the teeth, where any rubbing process of bone against the tooth is impossible. On the other hand, bones are splendid tooth-wearers. One need only look at the teeth of a dog which has habitually gnawed bones to be convinced that these organs would have been in a better state of preservation had they not been so used. But the ill effects do not stop at this. Splintered bones may wound the walls of the stomach and set up violent gastric inflammation, which may terminate fatally. Instances have been recorded of perforation of the organ by such bodies. Sharp bones, especially those of chickens, often become lodged in the throat or wedged between the teeth, from which they are removed with difficulty. Dogs have been destroyed by ignorant policemen and others, supposedly suffering from rabies, when they were merely making frantic attempts to dislodge bones from their jaws. Old dogs commonly suffer from impaction of the rectum caused by lodgement of bone-grit that has failed to become dissolved or digested in the stomach, when operative measures are necessary to effect removal of the obstruction. When it is considered necessary to feed bony matter, bone should be shaved by means of the machines in use by poultry-breeders and fed in this finely divided condition.

Referring to Question No. 6, most of the writers complained of the expense attached to the feeding of the commercial foods. A good many found fault with them on account of a tendency some of these foods exhibit to production of relaxation of the bowels.

In reply to No. 7, the majority feed vegetables sparingly, say one to three times weekly, while some believe in a bountiful vegetable diet. Beets, onions, cabbage, turnips, and potatoes are selected in about the order named. A few object to the use of potatoes, but surely this is unreasonable.

No. 8 has brought out numerous pointers, which the reader will no doubt be interested to hear:

Raw eggs soften the coats of St. Bernards.

Use plenty of salt in all food for dogs.

Always best to feed a dog according to climate.

Frequent feeding of puppies is the secret of success; avoid anything but bread and milk for at least three months.

A light feed before exercise or hard work, with a full feed a short time after.

Feed fish once a week for a change.

Feed bitches in whelp raw meat daily for two weeks before whelping.

Beagles do best on raw meat.

Food should be varied.

"Never have lost a puppy from distemper since I gave them meat from the first."

